# First-line serplulimab plus chemotherapy versus chemotherapy alone in small-cell lung cancer patients with brain metastases: a multicenter, prospective cohort study

**DOI:** 10.3389/fimmu.2026.1858418

**Published:** 2026-06-08

**Authors:** Wan Zhang, Xiaojun Yang, Hualin Chen, Xingxiang Pu, Reyihanguli Tuohetashi, Xuan Wu, Zhanhong Xie, Di Wu, Yongguang Cai, Shi Jin, Xuli Guo, Kaitao Yao, Yongfeng Chen, Guanming Jiang

**Affiliations:** 1 Department of Oncology, The Tenth Affiliated Hospital, Southern Medical University (Dongguan People’s Hospital), Dongguan, China; 2 Department of Pulmonary Oncology, Affiliated Hospital of Guangdong Medical University, Zhanjiang, China; 3 Department of Medical Oncology, Lung Cancer, Hunan Cancer Hospital, Changsha, China; 4 Department of Respiratory and Critical Care Medicine, The First Affiliated Hospital of Sun Yat-sen University, Guangzhou, China; 5 Department of Medical Oncology, Peking University Shenzhen Hospital, Shenzhen, China; 6 Department of Oncology, The First Affiliated Hospital of Guangzhou Medical University, Guangzhou, China; 7 Department of Respiratory and Critical Care Medicine, Shenzhen People’s Hospital, Shenzhen, China; 8 The Fifth Department of Medical Oncology, Central Hospital of Guangdong Provincial Nongken, Zhanjiang Cancer Hospital, Zhanjiang, China; 9 Department of Oncology, Cancer Hospital & Shenzhen Hospital, Chinese Academy of Medical Sciences and Peking Union Medical College, Shenzhen, China; 10 Department of Oncology, Huizhou Central People’s Hospital, Huizhou, China; 11 Department of Oncology, The Second Affiliated Hospital of Shantou University Medical College, Shantou, China

**Keywords:** brain metastases, immunotherapy, prospective cohort study, serplulimab, small-cell lung cancer

## Abstract

**Background:**

Immunochemotherapy is standard for small-cell lung cancer (SCLC), yet efficacy and safety of serplulimab for patients with brain metastases (BM) remain to be fully elucidated in routine clinical practice.

**Methods:**

In this multicenter, prospective cohort study, SCLC patients with BM who received first-line serplulimab plus platinum-based chemotherapy or chemotherapy alone were included. Primary endpoint was intracranial progression-free survival (iPFS). Secondary endpoints included extracranial PFS, systemic PFS, overall survival (OS), tumor response, and safety.

**Results:**

A total of 62 patients with SCLC and confirmed BM were included. Patients treated with serplulimab achieved an intracranial objective response rate of 78.57%. With a median follow-up of 22.17 months, serplulimab combined with chemotherapy demonstrated significant benefits across all survival outcomes. The serplulimab group demonstrated significantly prolonged median iPFS (8.93 vs. 6.37 months; log-rank *P* = 0.004) and OS (28.77 vs. 12.95 months; log-rank *P* < 0.001) compared with chemotherapy alone. Exploratory analyses revealed that the addition of cranial radiotherapy to immunotherapy significantly augmented survival benefits. Safety profiles were manageable, with no new safety signals identified.

**Conclusion:**

First-line serplulimab plus chemotherapy demonstrates robust intracranial efficacy and survival benefits with manageable safety in SCLC patients with BM. Integrating cranial radiotherapy appears to offer complementary clinical value, supporting this multimodal strategy as a viable therapeutic option.

## 1 Introduction

Intracranial metastases are one of the most common and devastating neurologic complications of systemic cancer. Lung cancer is the predominant primary tumor to metastasize to the brain, with brain metastases (BM) developing in 39% to 56% of patients (1). Notably, although small-cell lung cancer (SCLC) accounts for only about 15%-20% of all lung cancer, its highly aggressive biological nature and high incidence of BM pose a monumental challenge to prognosis (2). This is evidenced by the fact that approximately 10% of SCLC patients present with BM at initial diagnosis, and an additional 40%-50% will develop intracranial progression during the disease course (3, 4). The prognosis for these patients is exceptionally poor, with a median overall survival (OS) of merely 5 months, underscoring the urgent need for more effective therapeutic strategies (5).

The management of SCLC with BM has traditionally presented a formidable clinical challenge, heavily reliant on local radiotherapy strategies such as whole-brain radiation therapy (WBRT) and stereotactic radiosurgery (SRS) (6). While chemotherapy is the cornerstone of the systemic therapy, the efficacy is intrinsically limited by its suboptimal penetration across the blood-brain barrier (BBB), often resulting in inadequate intracranial disease control (6, 7). A paradigm shift was ushered in with the integration of immune checkpoint inhibitors (ICIs). Pivotal phase III trials, such as IMpower133 (atezolizumab, PD-L1 inhibitor) and CASPIAN (durvalumab, PD-L1 inhibitor), demonstrated that adding ICIs to first-line etoposide-platinum chemotherapy confers a statistically significant survival benefit, establishing this combination as the contemporary standard of care for extensive-stage SCLC (8, 9). However, the applicability of these findings to patients with BM remains a subject of debate. In these landmark trials, patients with active or untreated BM were largely excluded or underrepresented. Consequently, while subgroup analyses suggested a trend toward survival benefit, they lacked the statistical power to provide definitive evidence for this high-risk subpopulation.

Recently, the therapeutic landscape has evolved further with the emergence of serplulimab. The phase III ASTRUM-005 trial demonstrated that serplulimab as the first anti-PD-1 monoclonal antibody to succeed in the first-line extensive-stage SCLC landscape, significantly extending median OS to 15.4 months in combination with chemotherapy (10). Nevertheless, similar to previous trials, the specific efficacy and safety profile of serplulimab in SCLC patients with baseline BM remains to be fully elucidated in routine clinical practice. To address this critical evidence gap, we initiated this prospective, real-world study to evaluate the efficacy and safety of serplulimab combined with chemotherapy versus chemotherapy alone as a first-line treatment for patients with SCLC and baseline BM. The primary objective of this study was to evaluate intracranial efficacy (focusing on intracranial progression-free survival [iPFS]), while secondary objectives included the assessment of systemic/extracranial efficacy and safety. The findings from this study are anticipated to provide the prospective evidence urgently needed to guide clinical decision-making for this vulnerable and understudied patient population.

## 2 Methods

### 2.1 Study design and eligibility

This is a multicenter, prospective study evaluating the efficacy and safety of first-line serplulimab plus chemotherapy versus chemotherapy alone in SCLC with BM. Eligibility criteria included (1): adult patients (≥18 years) (2); newly diagnosed, histologically or cytologically confirmed SCLC (3); magnetic resonance imaging (MRI) confirmed BM (4); treatment with first-line chemotherapy with or without serplulimab. The exclusion criteria were (1): leptomeningeal disease in the absence of parenchymal brain lesions (2); insufficient clinical data (3); the presence of other primary malignancies. Patients were enrolled consecutively based on the eligibility criteria during the study period. The sample size was determined by the accessible patient population at the participating centers during the predefined enrollment window.

The protocol of this study was reviewed and approved by the central institutional review board at the lead center, Dongguan People’s Hospital (ethical approval no. KYKT2023-014), which was recognized and accepted by all participating centers. The non-interventional study utilized de-identified data, the requirement for obtaining written informed consent was formally waived by the ethics committee. All patient data were kept confidential and handled in accordance with the Declaration of Helsinki.

### 2.2 Treatment

In this prospective cohort study, treatment selection and administration reflected real-world clinical practice, arising from clinician-patient shared decision-making that considered physician discretion, standard guidelines, and individual patient preferences. Patients were categorized into two cohorts based on the systemic therapy they received in the first-line setting. The immunochemotherapy cohort was treated with serplulimab 4.5 mg/kg intravenously on day 1 of each 21-day cycle combined with etoposide + cisplatin/carboplatin (EP/EC). The chemotherapy cohort received similar standard chemotherapy regimens, consisting of etoposide 100 mg/m^2^ intravenously on days 1–3 plus either carboplatin AUC = 5 intravenously on day 1 or cisplatin 75 mg/m^2^ intravenously on day 1. The selection of the platinum agent, the actual number of treatment cycles (typically 4 to 6), and any necessary dose modifications were made at the discretion of the treating oncologist based on individual patient status, renal function, and toxicity profiles.

### 2.3 Assessment and endpoints

To ensure robust data quality, we implemented a structured collection process. All clinical information was retrieved from the electronic medical records system. Two trained investigators (Zhang W. and Yang XJ) independently populated the predefined electronic Case Report Form (eCRF) with the required baseline characteristics. The inter-rater reliability was assessed, and all discordant entries were flagged for review by a third investigator (Jiang GM), whose decision was considered final. Tumor response was assessed per the Response Evaluation Criteria in Solid Tumors (RECIST) version 1.1. Intracranial lesions were evaluated using MRI, while extracranial/systemic lesions were assessed using computed tomography (CT). During the first-line treatment phase, patients underwent radiographic evaluations every 6 weeks. Subsequent imaging assessments and survival status were monitored through routine outpatient records and proactive telephone follow-up. Adverse events (AEs) were assessed according to the National Cancer Institute Common Terminology Criteria for Adverse Events (CTCAE) version 4.0 from the initiation of first-line treatment until 28 days after the last dose. Events were followed until resolution, stabilization, loss to follow-up, or death.

The primary endpoint was iPFS, defined as the time since the initiation of first-line therapy until radiographic intracranial progression or death from any cause, whichever occurred first. Secondary endpoints included extracranial PFS, systemic PFS, OS, intracranial objective response rate (iORR) and disease control rate (iDCR), overall ORR and DCR, and safety. Extracranial PFS was defined as the time from the initiation of first-line therapy to the date of disease progression outside the central nervous system (CNS) or death from any cause. Systemic PFS was defined as the time from treatment initiation to disease progression (either intracranial or extracranial), or death from any cause. OS was defined as the time from initiation of first-line therapy to death from any cause. iORR and ORR were defined as the proportion of patients achieving complete response (CR) or partial response (PR) per the RECIST v1.1, for intracranial and systemic lesions, respectively. iDCR and DCR were defined as the proportion of patients achieving CR, PR or stable disease (SD) per the RECIST v1.1, for intracranial and systemic lesions, respectively.

### 2.4 Statistical analysis

Descriptive statistics summarized categorical variables as counts and percentages (n [%]), and continuous variables as mean ± standard deviation or median (range) based on normality tests. Survival outcomes were visualized using Kaplan-Meier curves. Differences between treatment groups in these survival endpoints were formally tested with the log-rank test, with results expressed as median survival times and 95% confidence intervals (CIs). Furthermore, we performed subgroup analyses across predefined baseline characteristics. Univariate and multivariate Cox proportional-hazards models were used within predefined subgroups to quantify associations between survivals and baseline characteristics, presented as HRs with 95% CIs. The tumor response rates were reported alongside 95% CIs, derived from the Clopper-Pearson method. A two-sided *P*-value of <0.05 was considered statistically significant. All statistical analyses were performed using R software (version 4.3.2).

## 3 Results

### 3.1 Baseline characteristics and treatment

A total of 62 patients with SCLC and confirmed BM were included in the study. Based on the first-line treatment regimen, patients were categorized into the serplulimab cohort (n=42) and the chemotherapy cohort (n=20). The baseline demographic and clinical characteristics of the study patients are summarized in **Table 1**. Overall, the two cohorts were generally well-balanced regarding baseline characteristics. The median age was 68 years in the serplulimab cohort and 58 years in the chemotherapy cohort (*P* = 0.054). The majority of patients were male (90.48% vs. 95.00%; *P* = 0.910) and had an ECOG performance status (PS) of 0-1 (90.48% vs. 90.00%; *P >*0.05). Furthermore, no statistically significant differences were observed between the two groups regarding smoking history (*P* = 0.258), concurrent comorbidities (*P* = 0.071), presence of liver metastasis (*P* = 0.608), bone metastasis (*P* = 0.506), or the number of intracranial lesions (*P* = 0.874). Notably, patients receiving serplulimab were more likely to present with brain-related symptoms at baseline than those receiving chemotherapy alone (42.86% vs. 15.00%; *P* = 0.030).

**Table 1:** Baseline demographic characteristics of included patients (N=62).

Variable, n (%)	Serplulimab cohort (n=42)	Chemotherapy cohort (n=20)	*P*
Age (years), Median (Range)	68 (39, 76)	58 (47, 74)	0.054
Sex			
Female	4 (9.52)	1 (5.00)	0.910
Male	38 (90.48)	19 (95.00)	
ECOG PS			
0-1	38 (90.48)	18 (90.00)	>0.999
≥2	4 (9.52)	2 (10.00)	
Smoking history			
Current/Former	31 (73.81)	18 (90.00)	0.258
No	11 (26.19)	2 (10.00)	
Comorbidities			
Yes	27 (64.29)	8 (40.00)	0.071
No	15 (35.71)	12 (60.00)	
BM-related symptoms			
Yes	18 (42.86)	3 (15.00)	0.030
No	24 (57.14)	17 (85.00)	
Liver metastasis			
Yes	12 (28.57)	7 (35.00)	0.608
No	30 (71.43)	13 (65.00)	
Bone metastasis			
Yes	14 (33.33)	5 (25.00)	0.506
No	28 (66.67)	15 (75.00)	
Number of intracranial lesions			
1-2	24 (57.14)	11 (55.00)	0.874
≥3	18 (42.86)	9 (45.00)	

BM-related symptoms were defined as neurological manifestations at baseline, primarily including mild to moderate symptoms such as headache, dizziness, nausea, and vomiting. No patients in either cohort presented with life-threatening neurological crises or severe functional impairment requiring urgent neurosurgical intervention.

Regarding treatment patterns, cranial radiotherapy was administered to 23 patients in the serplulimab cohort and 12 patients in the chemotherapy cohort. In the chemotherapy cohort, cranial irradiation was exclusively conducted within the first-line setting, with no patients receiving radiotherapy after progression. Conversely, 14 patients in the serplulimab cohort received cranial radiotherapy as part of the first-line strategy, while 9 patients received salvage radiotherapy after progression. In terms of radiation modality, all patients received WBRT therapy in the chemotherapy cohort, whereas the serplulimab cohort utilized both WBRT and SRS. Thoracic radiotherapy was administered to 7 patients in the serplulimab cohort and 2 patients in the chemotherapy cohort. In addition, 34 patients in the serplulimab cohort proceeded to maintenance treatment, while 5 patients in the chemotherapy cohort received maintenance therapy (**Supplementary Table 1**).

### 3.2 Survival

**Figure 1:**
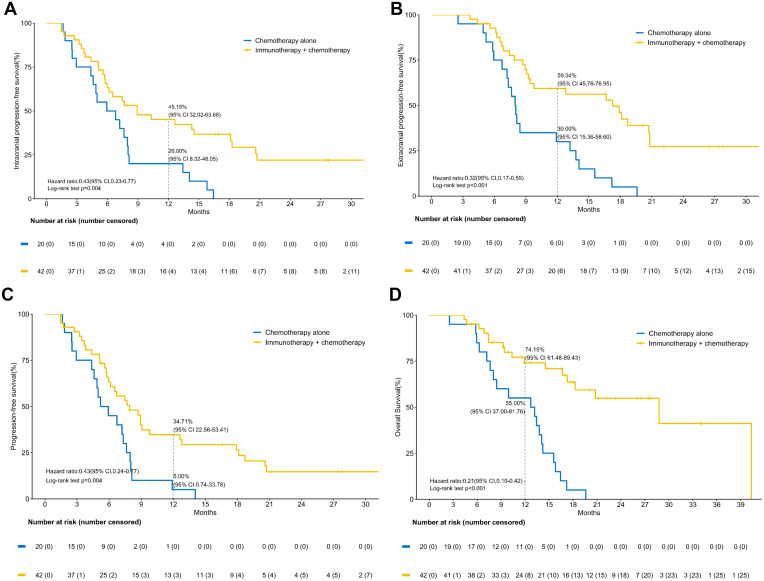
Kaplan-Meier estimates of survival outcomes in the overall population. Survival curves of **(A)** intracranial progression-free survival (iPFS); **(B)** extracranial PFS; **(C)** systemic PFS; and **(D)** overall survival (OS) in patients treated with first-line serplulimab plus chemotherapy compared to those receiving chemotherapy alone.

With a median follow-up of 22.17 months (range: 2.53-40.33), the combination of serplulimab and chemotherapy demonstrated significant benefits across all survival outcomes. Regarding the primary endpoint, the serplulimab cohort achieved a significantly prolonged median iPFS of 8.93 months (95% CI: 6.17-18.27) compared with 6.37 months (95% CI: 4.60-8.13) in the chemotherapy cohort (log-rank *P* = 0.004). This corresponded to a 57% reduction in the risk of intracranial progression or death (HR = 0.43, 95% CI: 0.23-0.77; **Figure 1A**), with 12-month iPFS rates of 45.15% and 20.00%, respectively.

Consistent improvements were observed in extracranial and systemic PFS. The median extracranial PFS was significantly extended in the serplulimab cohort to 17.27 months (95% CI: 9.40-20.80) versus 8.00 months (95% CI: 7.20-13.77) in the chemotherapy cohort (HR = 0.32, 95% CI: 0.17-0.59; log-rank *P* < 0.001; **Figure 1B**). Similarly, the median systemic PFS was significantly prolonged in the serplulimab cohort compared with the chemotherapy cohort (7.90 vs. 5.56 months, log-rank *P* = 0.004; HR = 0.43, 95% CI: 0.24-0.77; **Figure 1C**).

In terms of OS, the serplulimab combination conferred a substantial benefit. The median OS reached 28.77 months (95% CI: 17.27-not reached) in the serplulimab cohort, significantly exceeding the 12.95 months (95% CI: 8.03-15.57) observed in the chemotherapy cohort (HR = 0.21, 95% CI: 0.10-0.42; log-rank *P* < 0.001; **Figure 1D**).

### 3.3 Tumor response

Regarding intracranial efficacy, the serplulimab cohort exhibited a numerically higher iORR of 78.57% (33/42; 95% CI: 63.19-89.70%) compared with 75.00% (15/20; 95% CI: 50.90-91.34%) in the chemotherapy cohort. Similarly, the iDCR favored the serplulimab cohort (90.48% vs. 85.00%). Notably, a higher proportion of patients treated with serplulimab achieved an intracranial CR compared with those receiving chemotherapy alone (16.67% vs. 5.00%). Intracranial efficacy was further evaluated by stratifying according to the use of first-line cranial radiotherapy (**Supplementary Table 2**). Among patients who did not receive first-line cranial radiotherapy, the iORR was 75.00% in both serplulimab and chemotherapy alone cohorts. In patients receiving first-line cranial radiotherapy, the iORR was 85.71% in the serplulimab cohort and 75.00% in the chemotherapy alone cohort.

In terms of systemic efficacy, the serplulimab cohort achieved an ORR of 85.71% (36/42; 95% CI: 71.46-94.57%), numerically exceeding the 70.00% (14/20; 95% CI: 47.82-88.72%) observed in the chemotherapy cohort. The systemic DCR was comparable between the two treatment groups (90.48% vs. 90.00%). Detailed tumor response characteristics are presented in **Table 2**.

**Table 2:** Intracranial and systemic tumor response of included patients (N=62).

Tumor response	Serplulimab cohort (n=42)	Chemotherapy cohort (n=20)
Intracranial response		
CR	7 (16.67)	1 (5.00)
PR	26 (61.90)	14 (70.00)
SD	5 (11.90)	2 (10.00)
PD	4 (9.52)	3 (15.00)
iORR (95% CI)	78.57% (63.19-89.70%)	75.00% (50.90-91.34%)
iDCR (95% CI)	90.48% (77.38-97.34%)	85.00% (62.11-96.79%)
Systemic response		
CR	1 (2.38)	0
PR	35 (83.33)	14 (70.00)
SD	2 (4.76)	4 (20.00)
PD	4 (9.52)	2 (10.00)
ORR (95% CI)	85.71% (71.46-94.57%)	70.00% (47.82-88.72%)
DCR (95% CI)	90.48% (77.38-97.34%)	90.00% (69.62-98.83%)

### 3.4 Subgroup and multivariate analyses of survival

Subgroup analyses were performed to evaluate the consistency of survival benefits across prespecified baseline characteristics. For the primary endpoint of iPFS, the hazard ratios consistently favored the serplulimab combinations across various subgroups, a pattern of efficacy that was consistently observed across extracranial PFS, systemic PFS, and OS (**Supplementary Figure 1**). Notably, despite the higher baseline prevalence of brain-related symptoms in the serplulimab cohort, significant benefits in both intracranial and systemic PFS were maintained in symptomatic patients.

Additionally, multivariate Cox regression analyses were conducted to account for potential confounders. After adjusting for intracranial radiotherapy (Model 1), key baseline variables (Model 2), and all baseline characteristics (Model 3), the association between serplulimab-based immunochemotherapy and improved survival outcomes remained statistically significant and fully consistent with the primary findings of this study (**Supplementary Table 3**).

### 3.5 Impact of cranial radiotherapy

**Figure 2:**
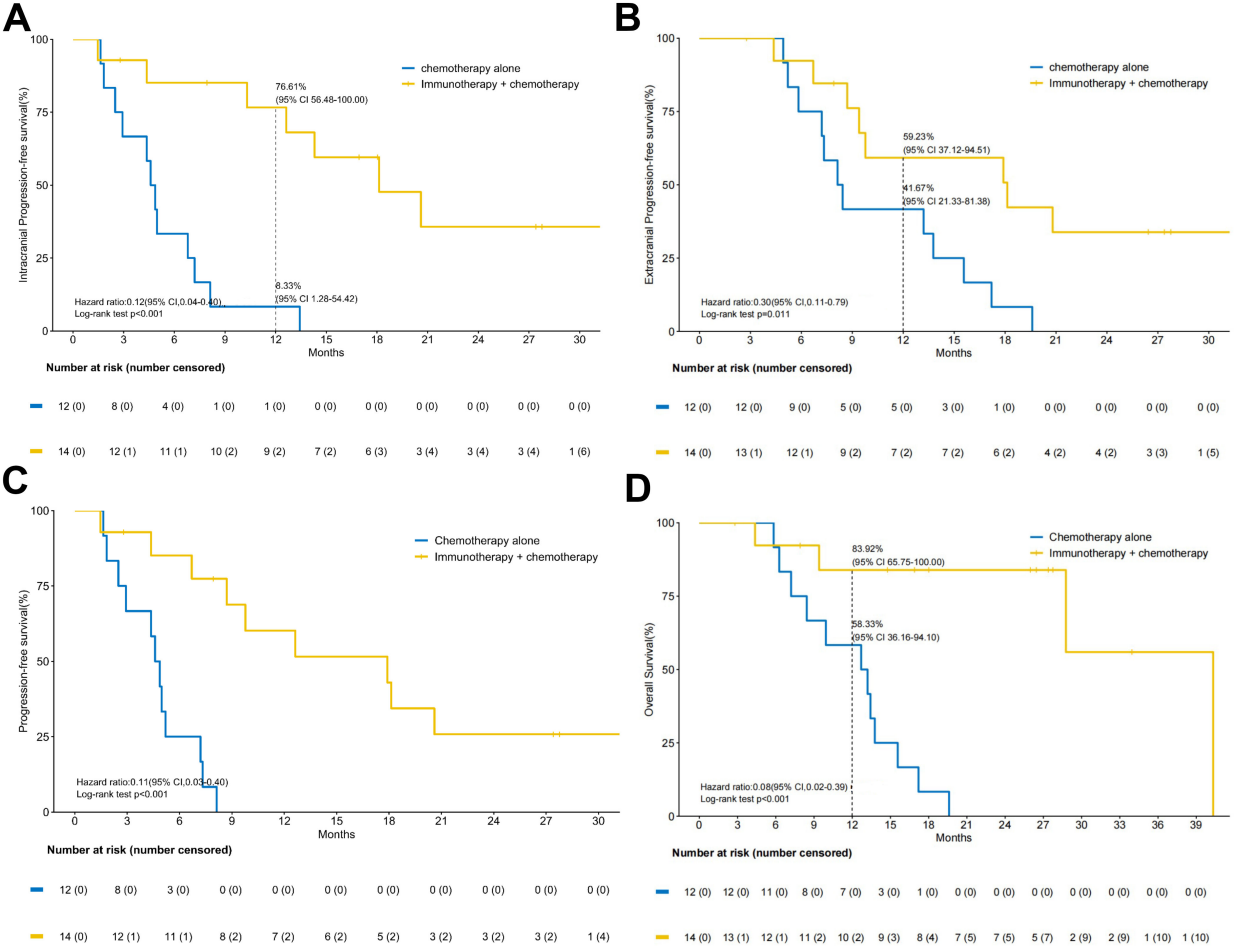
Comparison of treatment regimens in patients receiving first-line cranial radiotherapy. Survival curves of **(A)** intracranial progression-free survival (iPFS); **(B)** extracranial PFS; **(C)** systemic PFS; and **(D)** overall survival (OS). This analysis compares the serplulimab plus chemotherapy group versus the chemotherapy alone group among patients who received first-line cranial radiotherapy.

**Figure 3:**
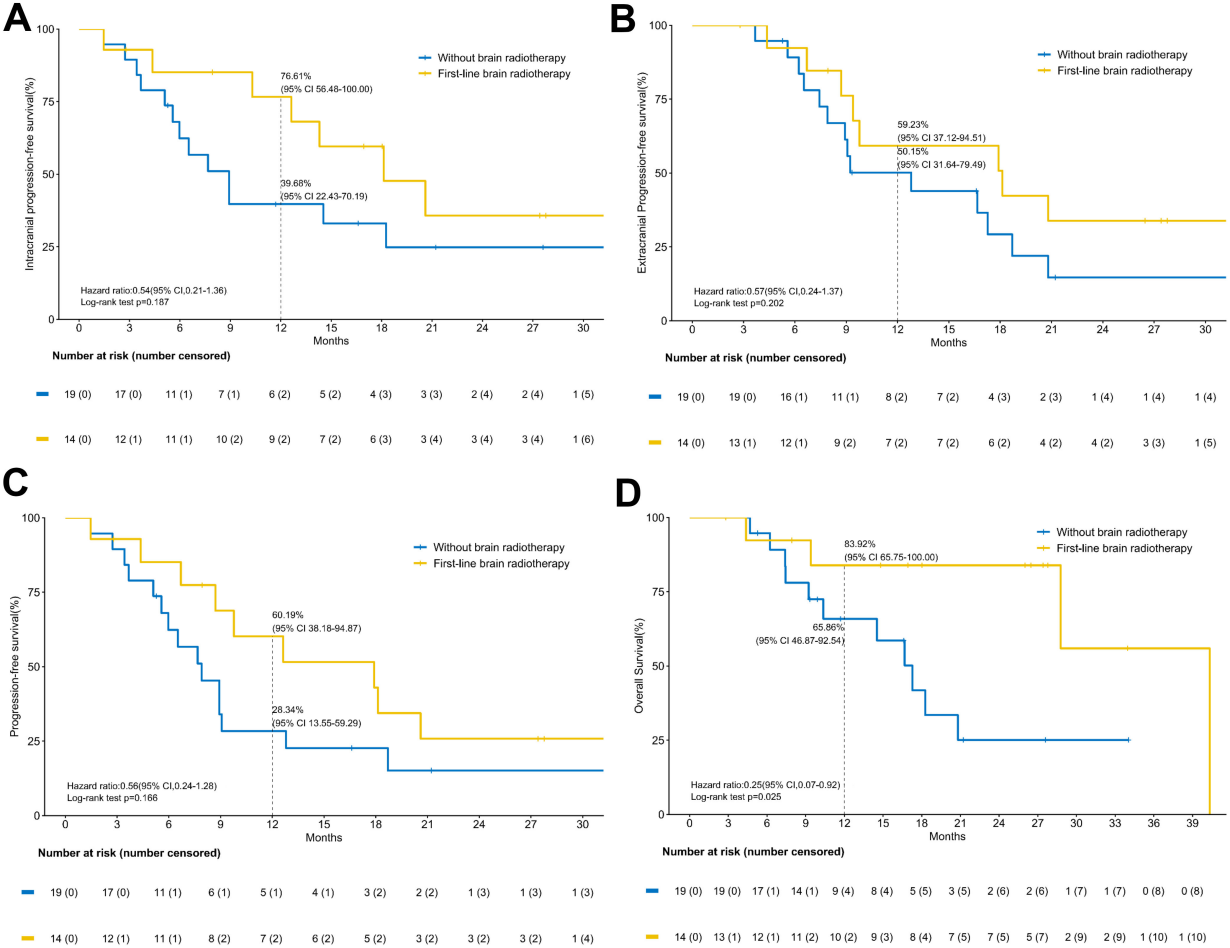
Comparison of first-line cranial radiotherapy status within the serplulimab cohort. Survival curves of **(A)** intracranial progression-free survival (iPFS); **(B)** extracranial PFS; **(C)** systemic PFS; and **(D)** overall survival (OS). This analysis compares patients who received first-line cranial radiotherapy versus those who did not, specifically within the cohort of patients treated with serplulimab and chemotherapy.

Given the pivotal role of cranial radiotherapy in the management of SCLC with BM, we performed exploratory analyses to explore its potential impact on survival outcomes. In the overall population, an unstratified comparison between patients receiving first-line cranial radiotherapy and those who did not showed no significant differences in PFS or OS (**Supplementary Figure 2**). However, stratification by treatment regimen revealed that the benefit of cranial radiotherapy was largely driven by the combination with immunotherapy. Among patients receiving first-line cranial radiotherapy, the addition of serplulimab to chemotherapy yielded significant improvements compared to chemotherapy alone, with significant benefits observed in intracranial (HR = 0.12; log-rank *P* < 0.001; **Figure 2A**), extracranial (HR = 0.30; log-rank *P* = 0.011; **Figure 2B**), and systemic PFS (HR = 0.11; log-rank *P* < 0.001; **Figure 2C**), as well as OS (HR = 0.08; log-rank *P* < 0.001; **Figure 2D**). Consistent survival benefits were observed in patients receiving first-line cranial radiotherapy compared to those who did not receive within the serplulimab cohort, further supporting the value of combining immunotherapy with cranial irradiation (**Figure 3**). Subsequent analyses of cranial radiotherapy timing and modality appeared influential with earlier radiotherapy timing (upfront vs. salvage; **Supplementary Figure 3**) and precision technique (SRS vs. WBRT; **Supplementary Figure 4**) were associated with better clinical outcomes within the combined modality approach.

### 3.6 Safety profile

The safety profile of the study population is summarized in **Table 3**. Treatment-emergent adverse events (TEAEs) of any grade occurred in 27 patients (64.29%) in the serplulimab cohort, compared with 8 patients (40.00%) in the chemotherapy cohort. Grade 3–4 TEAEs were reported in 10 patients (23.81%) in the serplulimab cohort and 3 patients (15.00%) in the chemotherapy cohort. A total of 13 patients (30.95%) experienced immune-related adverse events (irAEs), with 6 patients (14.29%) experiencing grade 3–4 events. Overall, the combination of serplulimab and chemotherapy demonstrated a manageable safety profile consistent with previous reports, with no new safety signals or grade 5 toxicity were identified.

**Table 3:** Safety profile of included patients (N=62).

Safety	Serplulimab cohort (n=42)	Chemotherapy cohort (n=20)
TEAE	27 (64.29)	8 (40.00)
Grade 1-2	20 (47.62)	5 (25.00)
Grade 3-4	10 (23.81)	3 (15.00)
irAE	13 (30.95)	–
Grade 1-2	10 (23.81)	–
Grade 3-4	6 (14.29)	–

## 4 Discussion

In this multicenter, prospective cohort study, we evaluated the clinical outcomes of first-line serplulimab combined with chemotherapy versus chemotherapy alone in SCLC with BM. Our findings indicated that serplulimab-based immunochemotherapy significantly extended intracranial and systemic survival outcomes. This substantial survival benefit was accompanied by durable intracranial control, suggesting that effective management of CNS disease via the combination strategy contributes to improved systemic outcomes. Our exploratory analysis indicated that these clinical benefits might be optimized by the integration of upfront cranial radiotherapy. These findings support the use of serplulimab-based regimens as a viable first-line therapeutic option for this challenging patient population.

Our study provides critical clinical evidence substantiating the intracranial efficacy of serplulimab plus chemotherapy in SCLC patients with BM. Regarding local control, while the initial iORR was numerically comparable between treatment groups due to inherent chemotherapy sensitivity of SCLC, the serplulimab-based combination therapy elicited a robust response intracranial CR rate (16.67% vs. 5.00%) and provided more durable survival benefits. This intracranial activity may be attributed to the compromised integrity of the blood-tumor barrier (BTB) caused by neovascularization within metastases, which facilitates the extravasation of macromolecules (11). Furthermore, the synergy between immunotherapy and radiotherapy may induce immunogenic cell death and remodel the tumor microenvironment, enhancing T-cell trafficking and BBB permeability (12, 13). Furthermore, a recent study has elucidated that anti-PD-1 immunotherapy can actively disrupt the BBB by inducing DKK1 expression in activated CD8+ T cells, a mechanism that not only facilitates the infiltration of therapeutic agents but also potentially accounts for the heterogeneous clinical responses observed in patients with BM (14). Our study demonstrated significantly prolonged survival benefits that compares favorably with historical data. While the CASPIAN trial suggested a trend toward reduced risk of new CNS lesions with durvalumab, the benefit failed to reach statistical significance, and the reported median iPFS of 4.7 months was numerically inferior to our finding (15). Furthermore, a recent phase II trial (LOGiK2001, SPEED) (16) evaluating first-line durvalumab plus chemotherapy specifically in SCLC patients with untreated BM reported an iORR of 66.7% and a median iPFS of 4.2 months. Similar systemic PFS and OS benefits were also observed when compared with the CASPIAN trial (15). In a real-world study evaluating the efficacy of first-line immunochemotherapy in extensive-stage SCLC patients with BM, atezolizumab-based or durvalumab-based therapy reported median OS ranging from 8.1 to 13.3 months and PFS from 4.63 to 5.33 months (17). This survival discrepancy likely reflects differences in patient selection and treatment modalities rather than drug efficacy alone. This real-world study included a higher proportion of elderly patients (>75 years) with potentially poorer performance status, whereas our study included a fitter population capable of tolerating intensive combined therapy. The integration of cranial radiotherapy in our study appears to be a key driver of this survival advantage. Furthermore, this advantage is potentially attributable to unique molecular features of serplulimab. Preclinical evidence suggests that serplulimab induces rapid PD-1 endocytosis and sustains T-cell activation via the CD28-dependent AKT pathway, while also favorably modulating the tumor microenvironment by reducing Treg cells and increasing effector T-cell density (18). These mechanisms may facilitate a more potent peripheral immune response and the subsequent migration of active effector cells into the CNS (14, 19). Additionally, the benefits with serplulimab were consistent across key predefined subgroups, underscoring the robustness of serplulimab in a representative, high-risk clinical population.

The promising efficacy observed in our study is likely rooted in the immunomodulatory effects of cranial radiotherapy, which may potentiate the activity of serplulimab. Beyond direct cytotoxicity, irradiation can induce immunogenic cell death and facilitate the release of tumor-associated antigens, effectively acting as an “*in situ* vaccine” (20). This process upregulates MHC molecules and presentation of novel antigenic determinants on the surface of cancer cells, thereby rendering them more susceptible to recognition by T-cell (21–23). Our study provides evidence supporting this multimodal strategy. Although both cohorts achieved an iORR of 75.00% within patients did not receive first-line cranial radiotherapy, the addition of cranial radiotherapy increased iORR to 85.71% only in serplulimab cohort. Furthermore, while unstratified analysis of first-line cranial radiotherapy in the overall population did not confer a survival advantage, stratification by treatment regimen revealed that its combination with serplulimab yielded pronounced benefits. Furthermore, within the serplulimab cohort, patients who received first-line cranial radiotherapy achieved superior survival outcomes compared to those who did not. These findings suggest that despite the systemic efficacy of serplulimab, local CNS control remains indispensable, and the combination of immunotherapy with cranial irradiation serves as a cornerstone for maximizing survival benefits in this population. Regarding the optimal radiotherapy strategy, our findings favor early intervention and precision techniques. This study indicated that upfront radiotherapy was associated with better clinical outcomes than salvage settings. This may be attributed to the rapid reduction of intracranial tumor burden, which prevents early neurological deterioration and preserves performance status, thereby creating a window of opportunity for the sustained efficacy of immunotherapy (24, 25). In addition, SRS demonstrated a trend toward superior outcomes compared with WBRT in our study. SRS allows for precise tumor ablation while sparing surrounding healthy tissue and minimizing systemic lymphopenia, thus potentially preserving the host’s systemic immune reservoir required for a robust anti-tumor response (26, 27).

These findings provide clinically relevant insights that may inform the management of SCLC with BM. First, our data offer prospective evidence supporting serplulimab-based immunochemotherapy as a viable first-line option for this high-risk population, complementing the outcomes of the ASTRUM-005 trial where specific intracranial data were less detailed. In addition, this study highlights the potential value of a multimodal strategy over systemic alone. The integration of cranial radiotherapy with serplulimab-based systemic therapy appears to provide a broader therapeutic window, likely by enhancing the local immune environment and optimizing intracranial control (28, 29). This approach addresses the dual requirement for rapid local response and sustained systemic protection, offering a practical reference for clinicians managing this challenging patient population in real-world setting.

Several limitations inherent to the observational design of this study warrant consideration. The non-randomized nature precludes the total elimination of selection bias and unmeasured confounding factors inherent to clinical decision-making in real-world settings. Baseline imbalances, particularly the higher frequency of symptomatic BM in the serplulimab cohort, may still influence outcomes despite multivariate adjustment for these potential confounders. The relatively high proportion of male patients in our study reflects the nature of consecutive enrollment in routine clinical practice; nonetheless, potential selection bias may exist despite the balanced gender distribution between the two cohorts. Furthermore, while investigators remained aware of the treatment regimens to facilitate safety monitoring and clinical management, the non-blinded radiographic evaluation might introduce assessment bias. The analyses regarding cranial radiotherapy modalities and timing were *post-hoc* and exploratory. The relatively modest sample sizes within these subgroups limit statistical power, necessitating that these findings be interpreted as hypothesis-generating rather than definitive. Although our study provides critical insights suggesting that first-line serplulimab-based immunochemotherapy improves clinical outcomes in SCLC patients with BM, the role of ICIs in this population warrants further formal documentation. Future prospective trials evaluating immunochemotherapy, particularly in combination with cranial radiotherapy, are essential to elucidate the full potential of immunotherapy and optimize its integration with radiation for the management of SCLC brain metastases.

## 5 Conclusion

This study provides compelling real-world evidence that serplulimab combined with platinum-based chemotherapy as first-line therapy confers significant survival advantages in SCLC patients with BM, alongside a manageable safety profile. Our data highlight the pivotal role of cranial radiotherapy in potentially amplifying this efficacy. These results support the adoption of this combination as a viable therapeutic option, and larger-scale, prospective trials are warranted to validate our findings.

## Data Availability

The original contributions presented in the study are included in the article/**Supplementary Material**. Further inquiries can be directed to the corresponding author.
