# Correction: First-line serplulimab plus chemotherapy versus chemotherapy alone in small-cell lung cancer patients with brain metastases: a multicenter, prospective cohort study

**DOI:** 10.3389/fimmu.2026.1922214

**Published:** 2026-07-20

**Authors:** Wan Zhang, Xiaojun Yang, Hualin Chen, Xingxiang Pu, Reyihanguli Tuohetashi, Xuan Wu, Zhanhong Xie, Di Wu, Yongguang Cai, Shi Jin, Xuli Guo, Kaitao Yao, Yongfeng Chen, Guanming Jiang

**Affiliations:** 1 Department of Oncology, The Tenth Affiliated Hospital, Southern Medical University (Dongguan People’s Hospital), Dongguan, China; 2 Department of Pulmonary Oncology, Affiliated Hospital of Guangdong Medical University, Zhanjiang, China; 3 Department of Medical Oncology, Lung Cancer, Hunan Cancer Hospital, Changsha, China; 4 Department of Respiratory and Critical Care Medicine, The First Affiliated Hospital of Sun Yat-sen University, Guangzhou, China; 5 Department of Medical Oncology, Peking University Shenzhen Hospital, Shenzhen, China; 6 Department of Oncology, The First Affiliated Hospital of Guangzhou Medical University, Guangzhou, China; 7 Department of Respiratory and Critical Care Medicine, Shenzhen People’s Hospital, Shenzhen, China; 8 The Fifth Department of Medical Oncology, Central Hospital of Guangdong Provincial Nongken, Zhanjiang Cancer Hospital, Zhanjiang, China; 9 Department of Oncology, Cancer Hospital & Shenzhen Hospital, Chinese Academy of Medical Sciences and Peking Union Medical College, Shenzhen, China; 10 Department of Oncology, Huizhou Central People’s Hospital, Huizhou, China; 11 Department of Oncology, The Second Affiliated Hospital of Shantou University Medical College, Shantou, China

**Keywords:** brain metastases, immunotherapy, prospective cohort study, serplulimab, small-cell lung cancer

In the published article, affiliation 1 was erroneously given as “Department of Oncology, The Tenth Affiliated Hospital of Southern Medical University (Dongguan People’s Hospital), Dongguan, China”. The original version of this article has been updated to reflect the correct affiliation: “Department of Oncology, The Tenth Affiliated Hospital, Southern Medical University (Dongguan People’s Hospital), Dongguan, China”.

There was a mistake in **Figure 2** and **Figure 3** as published. The images for **Figure 2** and **Figure 3** were accidentally swapped during the production stage. While the figure titles and captions remain entirely correct and in their proper places, the original image file displayed under **Figure 2** belongs to **Figure 3**, and vice versa.

**Figure 2:**
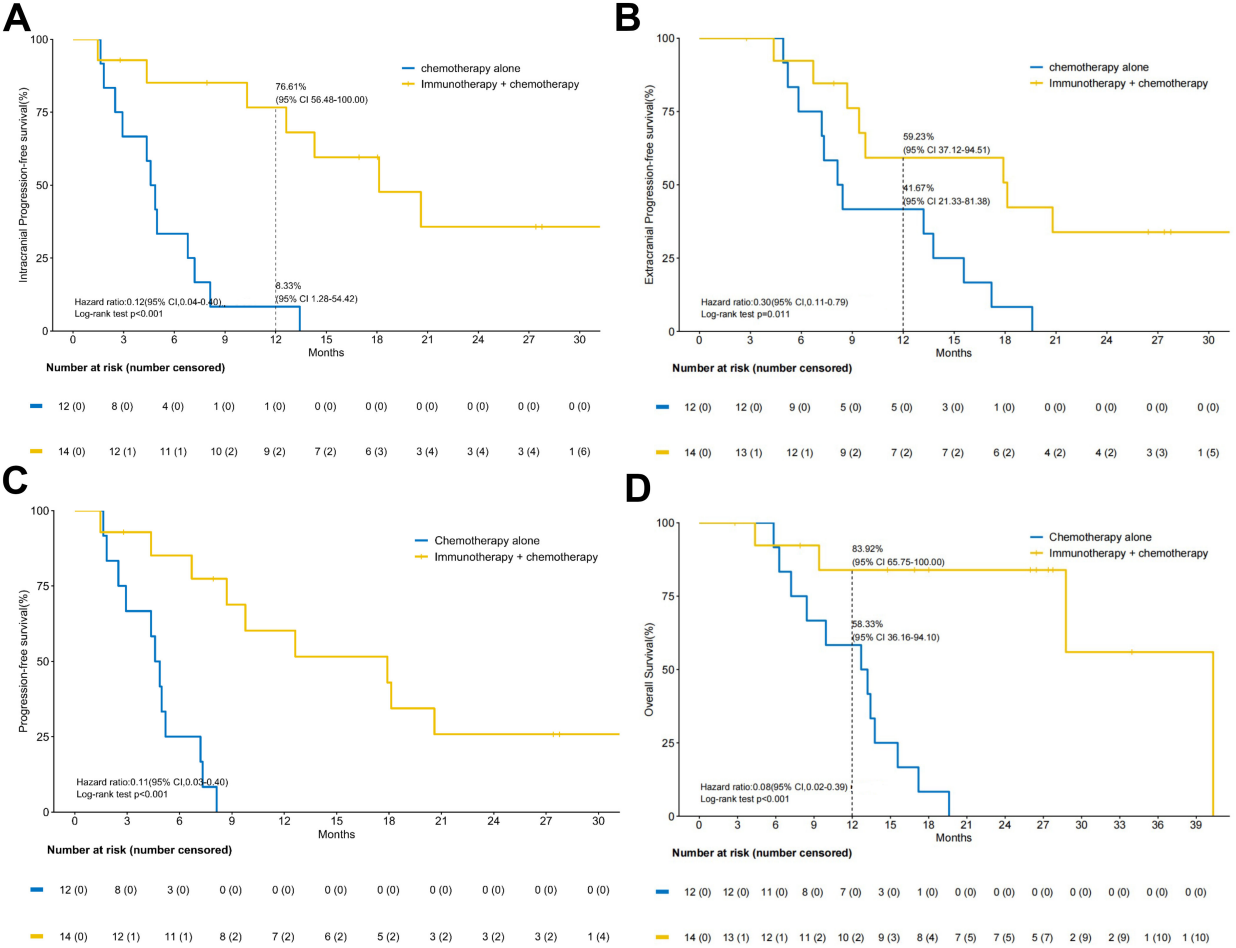
Comparison of treatment regimens in patients receiving first-line cranial radiotherapy. Survival curves of **(A)** intracranial progression-free survival (iPFS); **(B)** extracranial PFS; **(C)** systemic PFS; and **(D)** overall survival (OS). This analysis compares the serplulimab plus chemotherapy group versus the chemotherapy alone group among patients who received first-line cranial radiotherapy.

**Figure 3:**
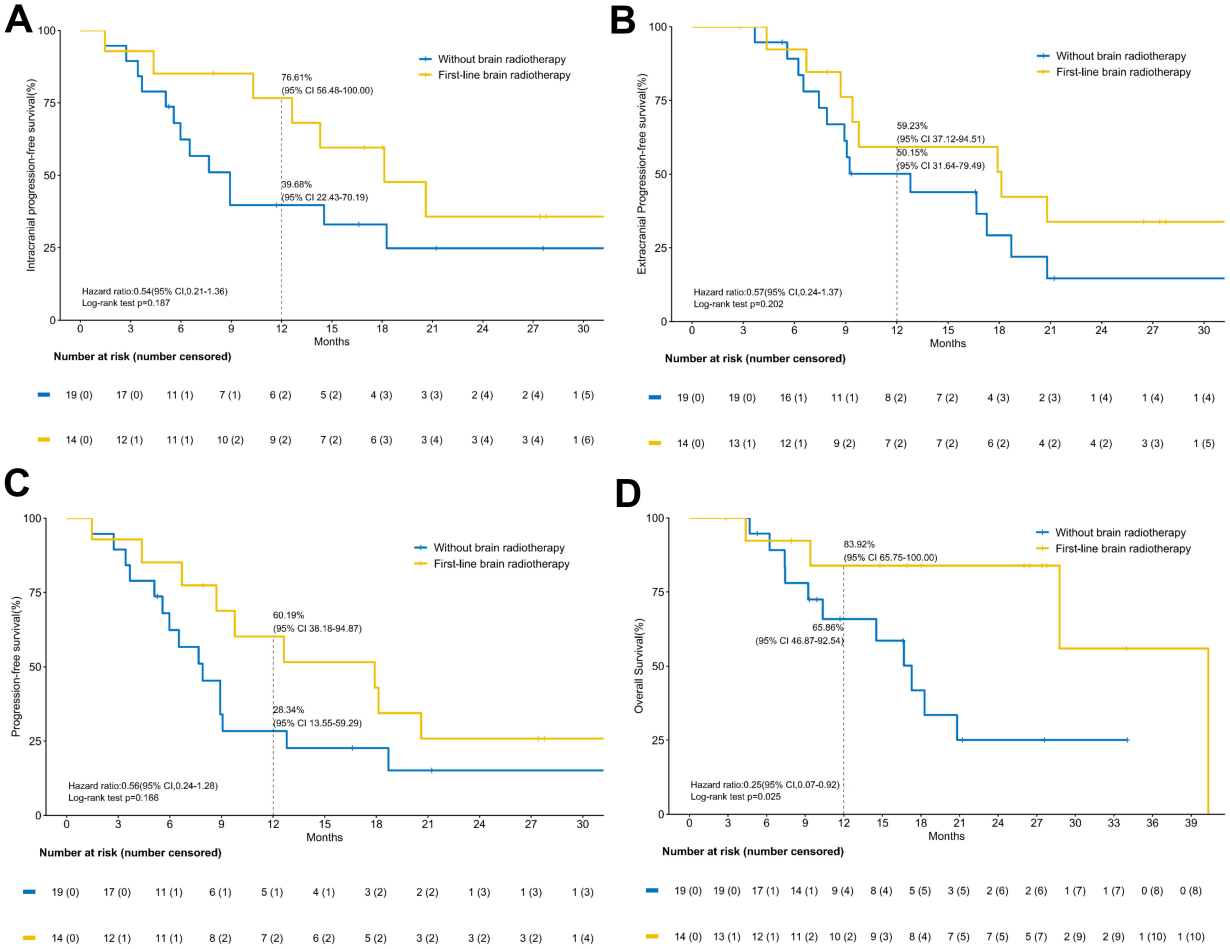
Comparison of first-line cranial radiotherapy status within the serplulimab cohort. Survival curves of **(A)** intracranial progression-free survival (iPFS); **(B)** extracranial PFS; **(C)** systemic PFS; and **(D)** overall survival (OS). This analysis compares patients who received first-line cranial radiotherapy versus those who did not, specifically within the cohort of patients treated with serplulimab and chemotherapy.

The corrected **Figure 2** and **Figure 3** appear below:

The original version of this article has been updated.

