# Supplementary material for: First-line serplulimab plus chemotherapy versus chemotherapy alone in small-cell lung cancer patients with brain metastases: a multicenter, prospective cohort study
Source: Front Immunol. 2026 Jun 8;17:1858418. doi: 10.3389/fimmu.2026.1858418 (PMC13284605; doi:10.3389/fimmu.2026.1858418)
Supplement: Supplementary file 1 [file DataSheet1.docx]

Supplementary Material

# Supplementary Figures


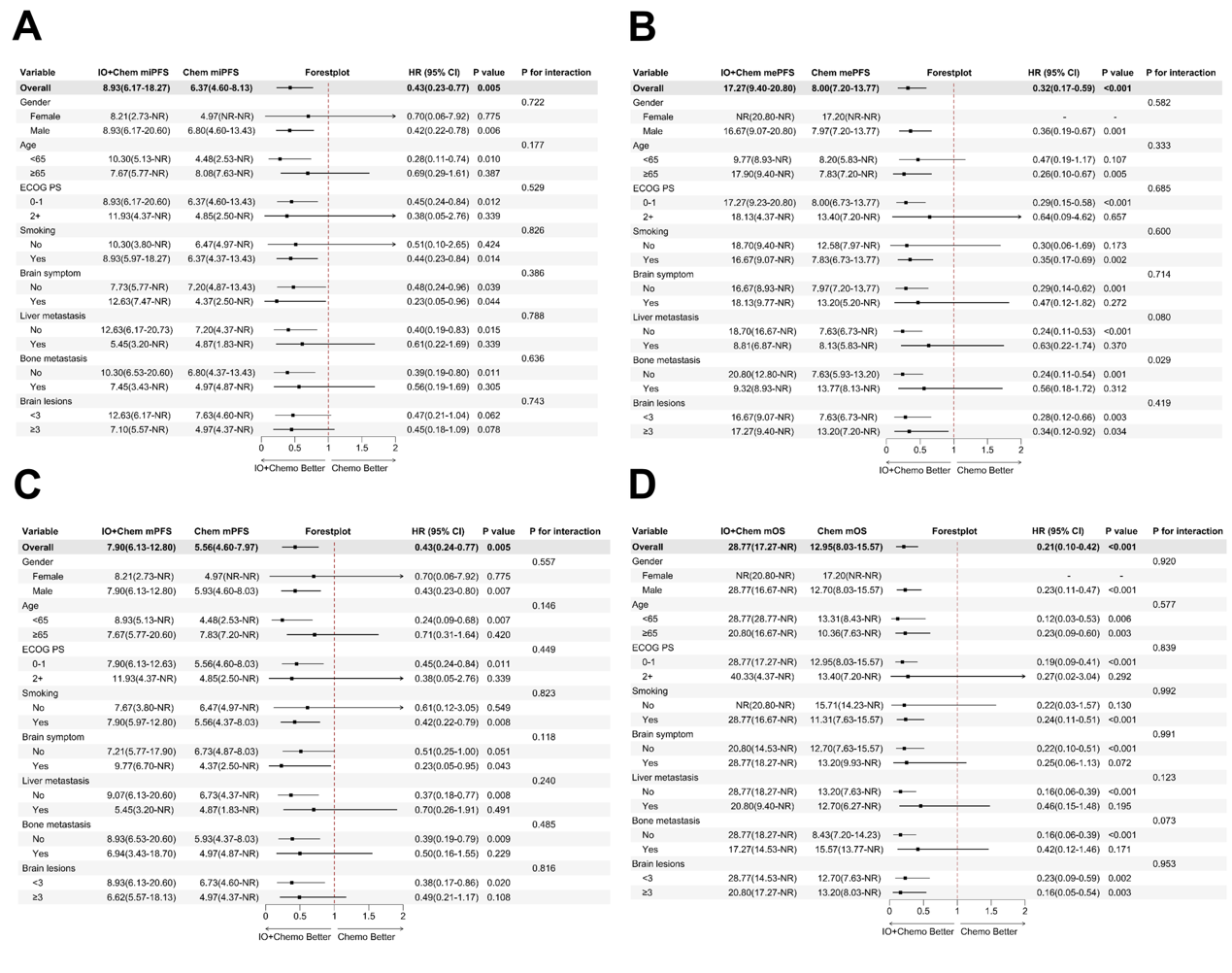
 **Supplementary Figure 1. Subgroup analyses of survival outcomes.**

Forest plots of (A) intracranial progression-free survival (iPFS); (B) extracranial PFS; (C) systemic PFS; and (D) overall survival (OS) across key prespecified baseline subgroups


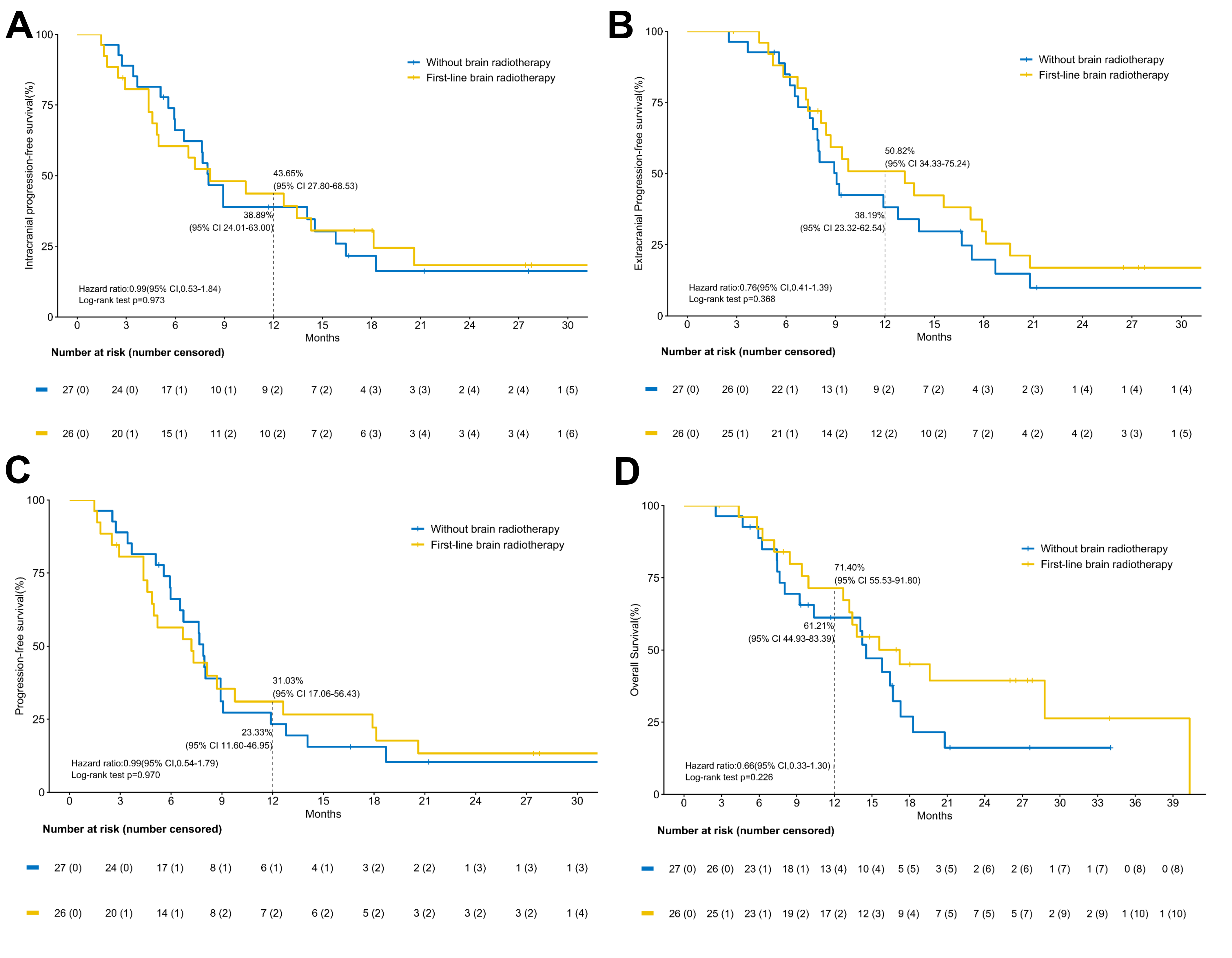


**Supplementary Figure 2. Survival outcomes based on cranial radiotherapy status in the overall population.**

Kaplan-Meier curves for (A) intracranial progression-free survival (iPFS); (B) extracranial PFS; (C) systemic PFS; and (D) overall survival (OS) comparing all patients who received first-line cranial radiotherapy versus those who did not, regardless of systemic therapy.


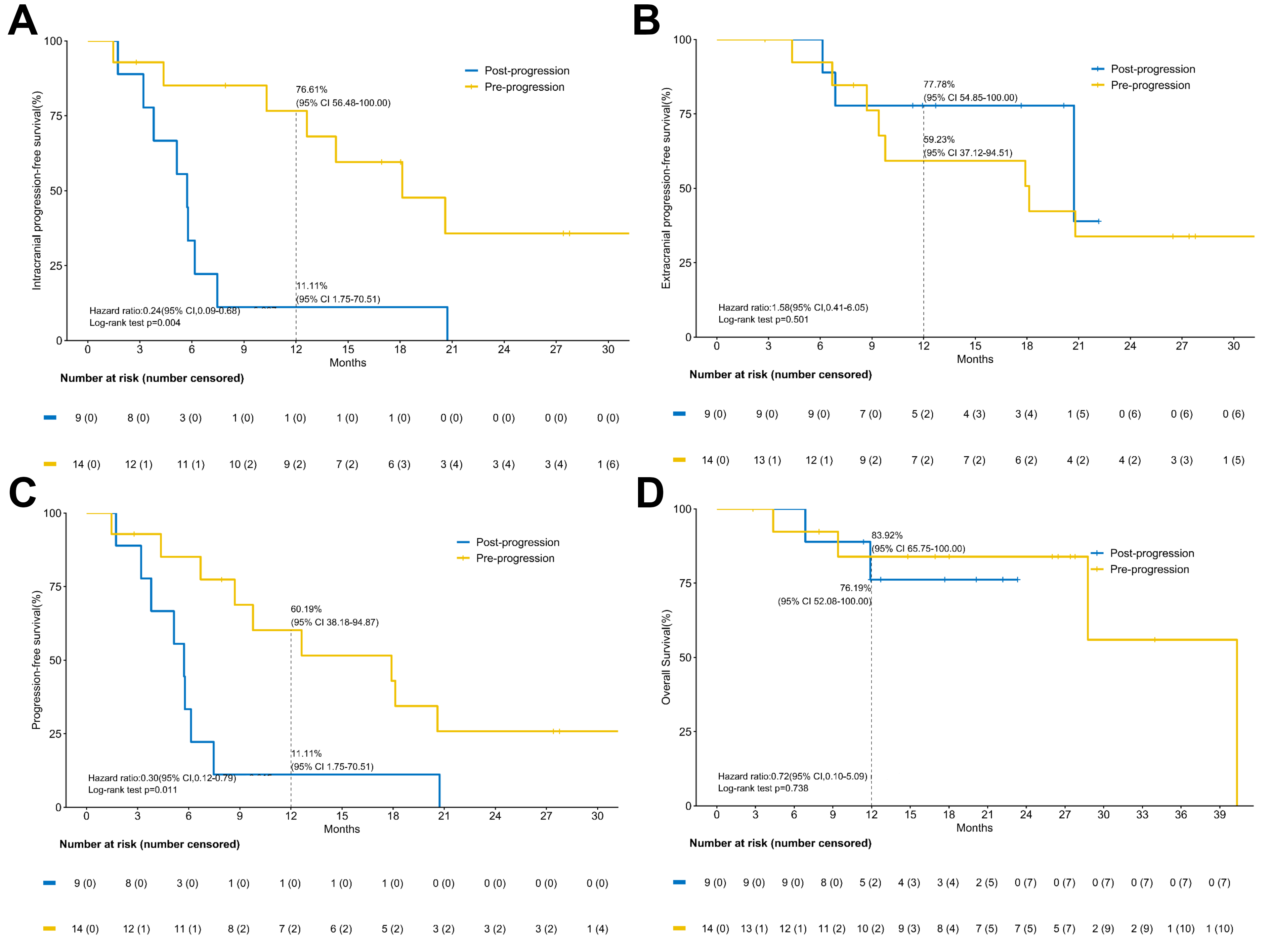


**Supplementary Figure 3. Impact of cranial radiotherapy timing on clinical outcomes.**

Exploratory analysis comparing (A) intracranial progression-free survival (iPFS); (B) extracranial PFS; (C) systemic PFS; and (D) overall survival (OS) in patients treated with upfront compared to salvage cranial radiotherapy.


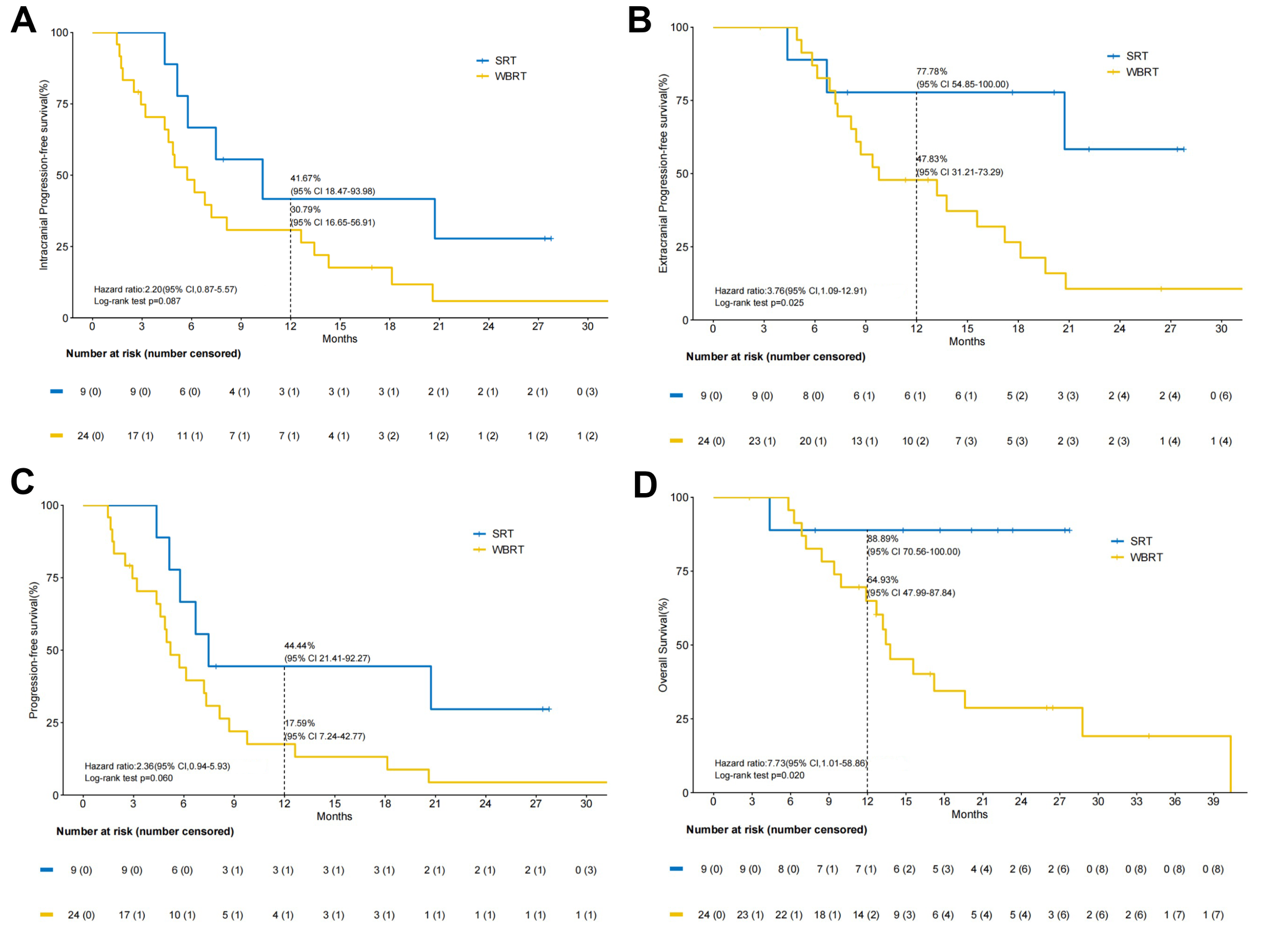


**Supplementary Figure 4. Comparison of cranial radiotherapy modalities.**

Exploratory analysis comparing (A) intracranial progression-free survival (iPFS); (B) extracranial PFS; (C) systemic PFS; and (D) overall survival (OS) in patients treated with stereotactic radiosurgery (SRS) compared to whole-brain radiotherapy (WBRT).

# Supplementary Table

**Supplementary Table 1. Treatment details of included patients (N=62)**

| **Variable, n (%)** | **Serplulimab cohort**  **(n=42)** | **Chemotherapy cohort**  **(n=20)** |
| --- | --- | --- |
| Cranial radiotherapy |  |  |
| Yes | 23 (54.76) | 12 (60.00) |
| No | 19 (45.24) | 8 (40.00) |
| Cranial radiotherapy setting |  |  |
| First-line | 14 (33.33) | 12 (60.00) |
| Post-progression | 9 (21.43) | 0 (0.00) |
| Cranial radiotherapy modality |  |  |
| SRT | 9 (21.43) | 0 (0.00) |
| WBRT | 12 (28.57) | 12 (60.00) |
| Unknown | 2 (4.76) | 0 (0.00) |
| Thoracic radiotherapy |  |  |
| Yes | 7(16.67) | 2 (10.00) |
| No | 35(83.33) | 18 (90.00) |
| Maintenance therapy |  |  |
| Yes | 34 (80.95) | 5 (25.00) |
| No | 8 (19.05) | 15 (75.00) |

**Supplementary Table 2. Intracranial tumor response stratified by first-line cranial radiotherapy**

| **Tumor response** | | **Serplulimab cohort** | | **Chemotherapy cohort** |
| --- | --- | --- | --- | --- |
| Patients who received first-line cranial radiotherapy | | | | |
| iORR (95% CI) | 85.71 (57.19-98.22) | | 75.00 (42.81-94.51) | |
| iDCR (95% CI) | 92.86 (66.13-99.82) | | 75.00 (42.81-94.51) | |
| CR | | 0 | | 0 |
| PR | | 12 (85.71) | | 9 (75.00) |
| SD | | 1 (7.14) | | 0 |
| PD | | 1 (7.14) | | 3 (25.00) |
| Patients who did not received first-line cranial radiotherapy | | | | |
| iORR (95% CI) | 75.00 (55.13-89.31) | | 75.00 (34.91-96.81) | |
| iDCR (95% CI) | 89.29 (71.77-97.73) | | 100.00 (63.06-100.00) | |
| CR | | 7 (25.00) | | 1 (12.50) |
| PR | | 14 (50.00) | | 5 (62.50) |
| SD | | 4 (14.29) | | 2 (25.00) |
| PD | | 3 (10.71) | | 0 |

**Supplementary Table 3. Multivariate Cox regression analysis of survival outcomes**

| Outcome | Model | HR (95% CI) | *P* |
| --- | --- | --- | --- |
| iPFS | Model 1 | 0.43 (0.24–0.78) | 0.005 |
|  | Model 2 | 0.50 (0.26–0.93) | 0.030 |
|  | Model 3 | 0.41 (0.19–0.88) | 0.022 |
| extracranial PFS | Model 1 | 0.28 (0.14–0.54) | <0.001 |
|  | Model 2 | 0.33 (0.15–0.71) | 0.005 |
|  | Model 3 | 0.34 (0.15–0.78) | 0.011 |
| systemic PFS | Model 1 | 0.43 (0.24–0.77) | 0.005 |
|  | Model 2 | 0.49 (0.26–0.92) | 0.026 |
|  | Model 3 | 0.40 (0.19–0.85) | 0.017 |
| OS | Model 1 | 0.18 (0.08–0.37) | <0.001 |
|  | Model 2 | 0.21 (0.09–0.48) | <0.001 |
|  | Model 3 | 0.23 (0.09–0.56) | 0.001 |

Model 1: Adjusted for cranial radiotherapy.

Model 2: Adjusted for cranial radiotherapy, age, comorbidities, liver metastasis, bone metastasis, and number of intracranial lesions.

Model 3: Adjusted for cranial radiotherapy, BM-related symptoms, sex, age, ECOG performance status, smoking history, comorbidities, liver metastasis, bone metastasis, and number of intracranial lesions.
